# Perspectives of people with spinal cord injury learning to walk using a powered exoskeleton

**DOI:** 10.1186/s12984-019-0565-1

**Published:** 2019-07-19

**Authors:** Patricia J. Manns, Caitlin Hurd, Jaynie F. Yang

**Affiliations:** Department of Physical Therapy, Faculty of Rehabilitation Medicine, University of Alberta, 3-48 Corbett Hall, Edmonton, AB T6G 2G4 Canada

**Keywords:** ReWalk, Qualitative

## Abstract

**Background:**

Powered exoskeletons for over ground walking were designed to help people with neurological impairments to walk again. Extended training in powered exoskeletons has led to changes in walking and physiological functions. Few studies have considered the perspective of the participants. The users’ perspective is vital for adoption of assistive devices. We explored the expectations and experiences of persons with spinal cord injury, training with the ReWalk exoskeleton.

**Methods:**

A qualitative research design with individual interviews was used. Eleven participants with spinal cord injury, taking part in 12 weeks of 4 times weekly training using the ReWalk, were interviewed before, immediately after, and 2 months after training. Interviews were audio recorded and transcribed verbatim. A six stage approach to thematic analysis was used.

**Results:**

The theme consistently expressed was the exoskeleton allowed participants to do everyday activities, like everyone else, such as looking people in the eye or walking outside. Their experiences were captured in three categories: 1) learning, a description of both expectations for learning and perspectives on how learning occurred; 2) changing, perspectives on perceived changes with training; and 3) contributing, which captured participant perspectives on contributing to research, including the giving of direct feedback regarding the exoskeleton (i.e., what worked and what could be changed).

**Conclusions:**

Incorporating the view of the user in the design and refinement of exoskeletons will help ensure that the devices are appropriate for future users. Availability and support for the use of exoskeleton devices in community settings is an interim step to home use as the devices continue to improve.

**Trial registration:**

www.clinicaltrials.gov (NCT02322125). Registered Dec 22, 2014 – Retrospectively registered after the first 4 participants had enrolled in the study.

## Background

The number of powered exoskeletons for training and restoring over ground walking in persons with neurological impairments has grown rapidly in the last decade (reviewed in Esquenazi et al., see reference [1]). Reports of exoskeleton safety and performance over a number of training sessions are emerging for a few devices including the ReWalk [2–6], the Ekso [7–11], and the Indego [12, 13]. Recent reviews [14–16] provide information about performance-based measures such as walking speed and endurance with exoskeleton walking. Walking speed is reported to be greater using an exoskeleton as compared to a reciprocating gait orthosis, but remains well below that required for community ambulation [14]. Walking endurance as reported from 14 studies with previously non-ambulatory participants with spinal cord injury (SCI), is a mean distance of 98 m during a 6 min walk test [15]. Some reports of physiological functions suggest improvements in bone density [17] and body composition [17, 18].

Given that adoption of an assistive device is highly dependent on the user’s perspective [19, 20], it is surprising that little research has focused on participant impressions of these powered exoskeletons and the learning process. A recent literature review [21] reported only 3 of 51 exoskeleton studies gathered user perspectives, and only 2 of the 3 were based on participant experience using the exoskeleton [3, 22], with the third study [23] based on impressions of the idea and potential of powered exoskeletons. Other studies have secondarily addressed topics with participants related to safety, ease of training, fatigue, effort during training, and effect of training in a ReWalk [2, 4, 24]. Participants expressed comfort in using the device after training, no increase in pain, but indicated that the learning process was not simple. The researchers suggested anecdotally that participants experienced emotional and psychosocial benefits [4], although this was not directly measured. In contrast, disability advocates argue that technologies only reinforce a focus on normalization, that in the end creates greater asymmetries between able-bodied and disabled [25, 26]. Research that seeks and incorporates feedback from people who will potentially use technological advances such as exoskeletons is critically important.

Qualitative methods, which provide a rich description of an experience [27] have been used in only one previous study to our knowledge to explore the perspectives of adults with SCI using an exoskeleton. Cahill and colleagues interviewed 4 non-ambulatory participants with SCI to learn about their use of an exoskeleton in a gym-based setting [28]. Participants discussed physical and social benefits of the exoskeleton and the adjustment of their thinking from the exoskeleton as a device to help them walk to one that allowed exercise through walking. They supported greater availability of exoskeleton technology outside of medical facilities. Additionally, a recent study used focus groups to solicit clinician experiences of exoskeleton training in 4 spinal cord injury centres in the United States [29]. Recommendations for facilities using exoskeletons in outpatient or wellness settings are described. The authors emphasize the gap in understanding of user perspectives on the costs and benefits of exoskeleton use and call for research in that area [29]. The paucity of studies that sought the user’s perspective motivated this qualitative study to explore the expectations and experiences of adults with SCI training to use the ReWalk exoskeleton. Further, since most individuals with SCI have limited access to such devices after training, we also explored their thoughts after they stopped training for some time.

## Methods

### Context

The method used in this study was qualitative description [30, 31]. The approach aims to “provide a rich description of the experience depicted in easily understood language” [32] and is used when the primary goals of the research are to learn from and describe participants’ experiences, and then utilize that new knowledge to guide future use of an intervention (e.g., an exoskeleton) [32]. The study was part of a prospective cohort study with persons with SCI (see companion paper), designed to test the functional gains and neuroplasticity induced by locomotor training with the ReWalk exoskeleton (ReWalk Robotics Ltd., Yokneam, Israel). The ReWalk is a wearable exoskeleton with battery-powered motors at the hip and knee that provide individuals with lower limb paralysis the ability to stand and walk. Participants trained using a Rehabilitation Model of the ReWalk exoskeleton 4 times per week over 12 weeks. They began by learning sit-to-stand and stand-to-sit transitions followed by balancing in standing. Walking training began once participants could maintain standing balance for > 30 s while lifting one crutch. Training consisted of practicing stopping and turning as well as walking on different surfaces such as linoleum, carpet, ramps, and outdoors on asphalt and grass. Each training session was 60–90 min long, with about 60 min of walking.

### Recruitment and interview procedures

Participants either self-referred or were recruited through a local non-profit agency (Spinal Cord Injury - Alberta), and through clinicians in the community who work with those with SCI. All participants provided informed written consent prior to participation (University of Alberta Health Research Ethics Board Pro00036789). Inclusion criteria were as follows: chronic (≥1 yr), non-progressive SCI, weight < 90 kg, thigh and lower leg length appropriate for the ReWalk, uses wheelchair as primary mode of mobility, able to use forearm crutches and train for 4 days/week, and approval from primary care physician to participate in the study. Exclusion criteria included: comorbidities that interfere with training or measurements, fractures within the last 2 years, bone density t-score < − 3, hip and knee contractures > 10° flexion, ankle plantarflexion contractures, active pressure ulcers, and severe spasticity.

Participants were interviewed prior to the start of training, immediately following training, and two months following training. An interviewer (PJM) followed a semi-structured interview guide (see Table 1) at each of the three time points. All interviews were audio recorded. Questions progressed from expectations and goals, to perspectives on training and its effects, to sustained effects at 2 months post-training. Interviews were 15–45 min in length, with the interview immediately after training taking the most time. The interviewer was not involved in the training of the participants, but was the assessor of the spasticity outcome reported in the companion paper.

**Table 1:** Semi-structured interview guide

Interview 1 - Pre-training 1. How did you find out about the ReWalk? Did you try to find out more about the ReWalk? How? 2. When you first saw or read about the ReWalk exoskeleton – what did you think? 3. What do you know about the exoskeleton (how it works, what’s involved in training, possible benefits)? 4. We all have some expectations when we start something new. When you think about training with ReWalk – what are your expectations about how easy or difficult it will be for you to learn to use the ReWalk? 5. What are your goals related to this ReWalk trial? What is the best outcome? What is the worst outcome?	
Interview 2 - Immediately post-training 1. What were your initial impressions of the ReWalk – once you had a chance to try it? How did it feel the first time you got up on your feet? 2. Tell me about the training. How did things progress over the 12 weeks? 3. Do you notice any changes since you started training? Explain (probe for details and timing of changes). 4. If we said today that you could take the ReWalk home with you, would you take it? 5. How did you feel as you came up to the end of training? 6. Follow-up on goals question from pre- intervention (remind participant of goals). Were goals reached, adjusted?	
Interview 3 - Two months post training 1. What have you been up to since training stopped? 2. Depending on previous responses ask – have the changes you discussed been sustained? Or have you noticed any changes in the last 2 months since training stopped?	

### Participants

Eleven people with traumatic spinal cord injury participated, 4 females and 7 males. One other participant (P6) completed baseline testing only and was not interviewed for the qualitative portion of the study. All participants used a wheelchair as their primary mode of transportation. Three participants were able to stand prior to training with support from a walking aid or a person. Two of them were able to complete a 6 min walk test using forearm crutches, the third was not able to take any steps even with assistance. The remaining participants could stand only with the use of a standing frame. Eight of 11 participants had interviews at all time points (pre-intervention, post-intervention, and 2 months post-intervention). Participant 5 stopped training after 6 weeks due to an injury sustained in a car accident and thus did not complete the full 12-week intervention. However, a post-intervention interview was done to ensure we learned about their experiences learning to walk with the ReWalk. Two others did not have a pre-intervention interview as the ethics approval for the qualitative portion of the study was finalized shortly after they started training. Participant characteristics are reported in Table 2.

**Table 2:** Participant characteristics

Participant	Age range	Time since injury (yr)	Mobility	Interviews
P1	2	4.2	NA	Post, Follow-up
P2	1	5.7	Assisted standing, walk short distance	Post
P3	1	2.5	Assisted standing	All
P4	3	24.2	NA	All
P5	2	16.2	NA	Pre and Post
P6	2	4.3	–	None
P7	1	2.4	NA	All
P8	2	1.3	NA	All
P9	1	2.0	NA	All
P10	2	4.4	NA	All
P11	3	18.7	Assisted standing, walk short distance	All
P12	1	1.6	NA	All
Mean* (SD)	37.5 (13.7)	7.8 (7.9)		

Age ranges are used rather than exact numbers to avoid potential identification of individuals. Age range: 1 = 18–30 yr., 2 = 31–50 yr., 3 = 50–65 yr. Mobility refers to any mobility in addition to wheelchair use; *NA* not applicable, participants used a wheelchair as their only mode of mobility

*Mean and SD do not include P6 who dropped out after 2 sessions of training

### Data analysis

All audio files were transcribed verbatim. The process of data analysis was iterative throughout the two-year data collection time period. The qualitative description approach focused on the participants’ awareness of their reality and their interpretation of events [32]. Use and reporting of verbatim quotes related to participant experiences are important and ensure that the analysis stays close to the literal description of events, while seeking a deep understanding of experiences [32].

**Fig. 1:**
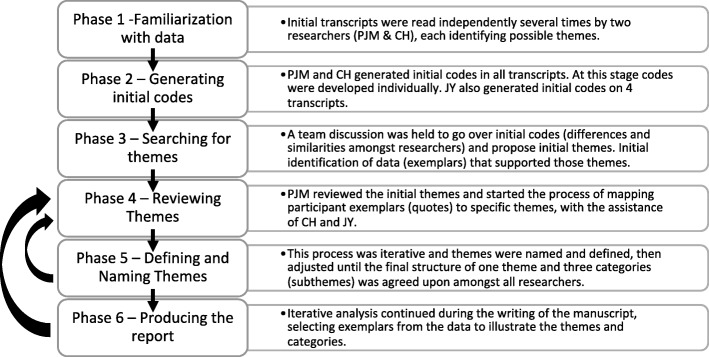
Data analysis phases and activities. Work on Phases 1–3 proceeded linearly. The processes related to phases 4, 5, and 6 were highly iterative as indicated with arrows. Phases are those recommended by Braun and Clarke (Reference [33]).

The process of analysis, as set out by Braun and Clarke, was used [33]. The six analysis phases are outlined in Fig. 1, along with our specific analysis activities related to each phase. Briefly, all transcripts were read independently by two of the authors, with the third author reading a subset (*n* = 4) of transcripts. All authors independently generated codes, which are small parts of the transcripts that related to preliminary concepts and possible themes (Phase 1 and 2 in Fig. 1). Codes and possible themes were discussed among the three authors (Phase 3). Mapping of quotes to initial themes, then generating the final theme and subthemes was an iterative process through Phases 4 to 6. There were frequent adjustments to theme/categories and their definitions as a result of analysis discussions amongst researchers. This process ensured theme/categories were appropriately grounded in the data (i.e., the interview transcripts). Throughout the analysis process, the definitions of themes and categories, as proposed by Morse, were used with the theme reflecting “a meaningful essence that runs through the data”, and categories which are a “collection of similar data sorted in the same place” [34]. By the time the final participant completed their interviews, it was clear that no new themes were being identified.

## Results

**Fig. 2:**
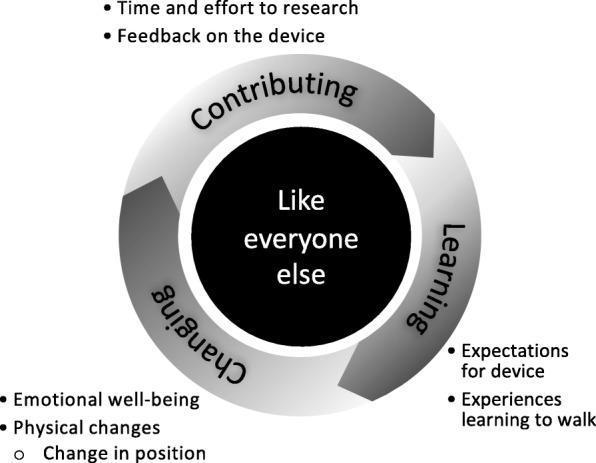
Theme and Categories

The process of data analysis resulted in one primary theme, like everyone else, along with three categories that together describe the experiences of persons with SCI taking part in exoskeleton training with the ReWalk. The theme and categories are displayed in Fig. 2.

### Theme – like everyone Else

One participant stated that they wanted to be “a regular upright”. This comment was made in the context of the need for bracing at the chest to assist them to stand upright. However, the comment embodies the theme that resonated throughout the conversations with participants, that they wanted to be able to do the same things as their peers. They felt the ReWalk allowed them to do everyday things like everyone else. Participants expressed joy in the experience of doing things such as walking outside, standing in the kitchen, or walking beside family. One participant reflected on the experience of walking outside, “…it was awesome - I kicked a few of the garden leaf piles over, stepped on a few bugs. Haven’t done that in a few years”. Several participants talked about how great it was to be back on the same level as others. A participant who has used a wheelchair for 19 years talked about that first time standing as an emotional experience, “it was the first time seeing eye to eye with someone instead of looking up.” The joy that the different perspective standing provides is exemplified in the following quote:

What a difference it makes from when you’re in a chair and you’re looking at stuff from 3 and a half feet from ground level with your eyes, to when you’re standing up. It’s like almost a whole new world out there, because it’s like night and day. Cause you’re focusing on what’s ahead of you 20 or 30 ft in your wheelchair, but you’re focusing down low. So when you’re standing up, of course you’ve got a way wider field of vision and depth, and so it’s way better – way more enjoyable

While the joys of training were linked to the ability to do everyday things, conversely, training was less enjoyable when it was unlike what others typically do. One of the younger participants stated, “it’s pretty boring just going back and forth [the hallway]”.

The many discussions about the importance of having a purpose and how ReWalk training provided that, allowed participants to feel like a regular person with a job to do. One stated,

I’m thinking most of the emotional benefits I had were from having a reason to get up and go somewhere. And you know, I guess having a purpose. You come here you do your guinea pig thing, and that’s my job. I think most of the emotional benefits came from that more so than the actual walking

In reflecting on stopping training, they further stated, “it feels like I’m getting fired or something”.

### Categories

In addition to the theme, there were three categories that describe the participants’ perspectives on training with the ReWalk including learning, changing and contributing. Each category is defined and explained below.

#### Learning

Participants talked about their learning experiences during the training including their expectations for learning, their perspectives on *how* they learned to use the ReWalk, and how easy or hard learning was. When asked about expectations for learning and training, participants often had vague goals or said they had “no expectations” or “low expectations.” One participant stated, “I didn’t really have any expectations, I was just happy to be here”. Another echoed, “happy to be part of the program”. Others stated that they didn’t want to commit to goals before experiencing the machine. The majority of participants indicated that they had not done much exploring on the internet to learn more about ReWalk. When participants did have expectations for training, they were generally physical in nature. These goals included things such as improved balance, increased strength, and confidence standing. Several participants talked about walking as a goal but often this was a long term hope, something that was a part of their everyday consciousness regardless of the ReWalk training, “the walking right? Everyone is dreaming of it”. The two individuals who were able to walk prior to training in the ReWalk both said that their goal was to increase their stamina for walking outside of the exoskeleton.

Once learning began, participants described their first impressions of the ReWalk, with one saying, “it’s kind of scary and at the same time, exciting, ‘cause you never know what can happen”. Another participant said,

It definitely wasn’t as comfortable or I wasn’t as stable, and I definitely wouldn’t say fear – but common sense and fear sometimes interfere with my plans. So they were knocking at the door when I got up there the first time, and I was like whoa man this isn’t going to be as easy as I thought

Two participants with complete SCI and one with incomplete SCI talked about the experience of learning to walk with the ReWalk as analogous to riding a bike. “Once you get it, you’ve got it”. The participant went on to say, “once you found those exact little, little movements, you were good to go”. An older participant discussed his learning process over the training period, “at the end it was much more finesse than it was a strength issue anymore, it was just there – you do learn a technique but it’s difficult for them [the trainers] to actually convey to you what that technique is. And you almost have to learn it by trial and error…” He went on to say, “after you get through the first part, then it starts to become more fun”. Other comments suggest that learning is retained once a level of walking proficiency is reached. One participant discussed coming back after a three week absense, “Maybe it was good to take a break, give your brain time to go over all that stuff, process it, right? Then when I came back, it was easier”. Several participants experienced periods of time during the training, when they could not train. Generally, participants had minimal or no loss of ability with a training break, based on quantitative measures of walking (see companion paper).

The amount of effort required during the learning process was also noted, not just physical but also mental. “It didn’t just walk on its own” was one of the initial comments of a participant as they learned to adjust to the machine and the movements needed to facilitate walking. This experience may have been more difficult for that particular participant as they did some walking at home with a walker, and may have also had to unlearn their current walking techniques (i.e., without the exoskeleton). The amount of effort required lessened as learning occurred, as expressed by one participant with complete SCI, “…probably exerted as much effort in that first day taking those ten steps at a time as I did when I did the 900”. Several participants talked about the effort required of the arms, “my arms getting used to it. ‘Cause obviously I wouldn’t be using those muscles as much, just wheeling”.

#### Changing

Participants were specifically asked about any changes they perceived occurring during training or in the two months after training stopped. While they talked frequently about emotional/mental health changes, participants often needed verbal probes to reflect on physical changes. We specifically asked about spasticity, pain, and bowel and bladder function. Four participants noticed a decrease in their spasticity, two of whom were able to reduce their dose of an antispasmodic medication while training. Two participants said their neuropathic pain decreased while training and both participants mentioned that the pain had been coming back over the two months following the end of training. Another physical change that occurred in a few participants with complete SCI was improved bowel routines. Three participants said that walking in the ReWalk led to faster and more regular bowel movements. One indicated that change was maintained two months after the end of training. Additional physical changes mentioned included improved circulation. One participant said, “I started to feel like I’m not cold as much all the time” and another two participants noticed improved sensation, one in their foot and the other described feeling hungry for the first time since their injury.

A second aspect of the discussion of physical changes was that training in the ReWalk provided an opportunity for a change of position, which was regarded as a positive thing; “just getting up and actually doing something different other than sitting down”. Change in positions was described as analogous to what people who walk do when they get home after work, “the first thing you want to do when you get home is go sit down. You’d be like I need to change positions, right. So that’s what it’s like... So [the ReWalk is an] opportunity to have something like that in day-to-day life”. In fact, one participant described the ability to stand easily as the best part of training.

Discussions of changes to mental and emotional well-being were frequent and interspersed throughout the interviews, often without specific prompting. Overall, participants found that their participation in the ReWalk study enhanced their mental well-being. They spoke of their time training in the ReWalk as encouraging saying “it gives you more hope” and “I feel tall and much happiness inside”. Another participant stated “it’s definitely helped me mentally as well as physically, it’s boosted confidence and it’s brought back this joy to my life”. Another stated, “to be able to go out in the sun and go for a walk – it’s unbelievable what that does for you”. These expressions of joy were sometimes contrasted with disappointment and sadness at the end of training. When reflecting on the first two months after training had ended, a participant said he felt “greedy” and wanted more. He went on to say “I walked again with ReWalk and then just sat for two months or three months at home, – it’s totally [laughs] you can say – it doesn’t feel good”.

The benefits to mental well-being over the course of training were not solely due to regular exercise or the opportunity to change positions. In fact, there was much discussion about the positive mental effects of having regular social interaction and being part of a community. This benefit was expressed by many participants. For example, one older participant, who reported that they typically spend a lot of time on their own, spoke of the benefit of regular social interaction saying “some of the other benefits that have come from this too are just the interaction with the people that were running the program… I feel like I became friends… because they were there every day”. This sentiment was echoed by one of the younger participants who remarked that “if you actually talk to [the trainers] like the way I was able to talk to them about stuff, you can connect with them, everything else just goes out the window”. When asked if they missed the walking two months after training had ended, one participant responded saying “Actually I do, part of it’s the company… like I really enjoyed working with [the trainers]”.

#### Contributing

When asked about their motivation to participate, the majority of participants talked about the importance of giving back and contributing. They talked about how glad they were to share their time, their efforts, and their user’s perspective and feedback of this new technology. Individuals were keen to participate in this study even if there was going to be no direct benefit to them. Before beginning training, one participant said “Even if it doesn’t benefit me, if I go through the study and you take all the notes, it’s going to benefit someone else and it’s going to benefit your research”. Another, when talking about the reasons for participating in the study, said “I just want to help. And this is how I can help, ‘cause I just donate myself to science”. Another said “I was thinking it was leading to something that would be practical down the road for someone. It was more a stepping stone than being practical for me, and me getting a huge amount of benefit out of it”. Part of contributing, and the happiness to contribute, seemed to be linked to the promise of technology to allow participants to do things they used to do as a “regular upright”. One participant stated, “I’ll just keep throwing myself under the bus. Yeah, go ahead inject wires into me, do whatever you want, eventually something - they’re going to come up with something real big, then I’ll be rocking and walking”.

Following the training period in the ReWalk, participants contributed to our knowledge of the device by reflecting on its usability, including how they would use it if they were to take it home. Nine of eleven participants said they would take the ReWalk home if the cost of the machine was not a factor. These individuals often responded enthusiastically saying “I’d grab it and go”, “I would love it” or another “Yeah I will. I know it’s helped a lot for me”. The participants who said they would take the ReWalk described the various ways in which they would use the device at home. Some said they would use it to walk outside, others mentioned it would be useful for chores or jobs that required standing and three participants specified that they miss being able to stand while cooking and that the ReWalk would be useful in the kitchen. One participant specified that they would use it at a community centre or gym where there is access to a track because there isn’t enough space to walk with the ReWalk in their home. A participant with an incomplete injury who walked independently prior to training in the ReWalk, envisioned using the device to increase the intensity of walking at home, saying “I’d like to be able to do those distances, just to push myself and get stronger, but I don’t have the means at home. Whereas if I had [the ReWalk], I think I could”. Other more specific feedback about the exoskeleton was related to wanting to be able to walk without a backpack unit, as well as streamlining and making the exoskeleton itself less bulky. Some of these improvements are already in progress and in fact were released during the trial. In general, participants felt like the device wasn’t ready for home use. For example, one participant commented, “it’s not ready for individual use. The user interface needs to be worked on”. Another participant stated, “it’s a pretty primitive machine and isn’t ready for home use, …will there down the road be a version of it that might be? Maybe.”

Though most participants said they would take the ReWalk home even though they couldn’t use it by themselves (i.e., must be used with a trained companion for safety), the two participants who said they wouldn’t take the machine did so because they needed assistance to safely ambulate in the device. One participant went on to clarify by saying “There’s no way I would feel confident enough to use it by myself, I need a spotter behind and in front… I don’t think I’d use it at home but I’d like to have access to one”. The second participant who said they would not take the device home also said that they would like to have access to one in a rehabilitation setting “It’s a good system… if it was just like physiotherapy you went to a location and they had a machine and you walked in it for an hour for exercise”.

## Discussion

### Like everyone Else

Learning about user experiences is a critical yet infrequently reported component of exoskeleton design, training and use. User perspectives impact and inform the translation of technological advances into clinical practice and ultimately everyday use [35]. This study provides the first in depth qualitative report of the experience of people with SCI during and following exoskeleton training. The theme that was pervasive was the participants’ reflections that the ReWalk allowed them to fulfil everyday roles, do everyday activities, and be a little more like everyone else. Participants talked about looking people in the eye, and about the different and enriched perspective on the environment when in an upright position. One participant talked about seeing everything from “a proper point of view”. These perspectives mirror the messages in the classic phenomenological manuscript of Erwin Straus [36] who states “upright we are, and we experience ourselves in this specific relation to the world”. (p532) He further states “that upright posture is an indispensable condition of man’s self preservation”. Recently, these assertions have been explored through a disability studies lens [37] but clearly our participants desired to be in an ‘upright’ world and saw an exoskeleton as a device that may help get them back there. They confirm the dominant discourses about walking (normal/abnormal distinction; walking is better) [38].

Our theme, ‘like everyone else’, is similar to the primary theme from a qualitative study by Jordan and colleagues who interviewed 5 adults with incomplete SCI taking part in locomotor training (using body weight support treadmill training) [39]. Participants in that study discussed the social significance of the physical change brought about by locomotor training and the struggle to attain normalcy in life activities. One participant stated, “he wanted to return to activities he enjoyed doing before the injury” [39]. By contrast, following Lokomat training, children with cerebral palsy stated that it wasn’t that important for them to walk normally [40]. This difference of opinion between adults and children, and the acquired (SCI) versus innate (cerebral palsy) nature of the conditions may support the view that disability is socially constructed [41] and that as we age we learn and understand more about what society considers ‘normal’. This finding may also simply reflect that children are accustomed to looking up and the difference when standing using technology is not as significant. Adults are used to looking people in the eyes prior to their injuries. These age and condition specific factors will be important for manufacturers of technological devices to consider going forward.

### Disability and technology

What message does our theme convey to designers of technological devices such as the ReWalk? Certainly, our participants were interested and excited about the promise of technology. They, like others with SCI, seemed to believe that ‘technology was on their side’ [39]. They were willing to invest their time training in devices they perceived allowed them to have ‘normal’ everyday experiences. How do we reconcile the views of our participants with the view from disability theorists that “technologies working within an order of normal are implicated in the (re) production of the asymmetries that they and it seek to undo” [25]? Do technologies provide only temporary respite and much sought after glimpses of normality, and then eventually reinforce asymmetries for participants? These are tensions that developers must be aware of and responsive to. In the future, a greater voice from persons with disabilities in the design and testing of technologies will increase our understanding of the role they can play, the health or functional benefits that may result from their use, and of their potential adoption.

### Reflections on learning, changing and giving

The experience of participants was overwhelmingly positive - though it was not only about the walking. It was about learning, about being with other people, about being able to contribute, and for some about having a job (a place to go 4 days a week).

Three participants used the analogy of bike riding to describe their learning process. Bike riding is often used as an example of a task that is learned implicitly, and participant comments about the learning process suggest that learning to walk with the ReWalk may be largely implicit. The observation that participants consistently and quickly regained their ability to walk with the ReWalk despite breaks during the training, including the 2 months following training, supports the implicit nature of learning. Future trainers should be aware of this and can limit the verbal instructions provided to new learners while supporting and reinforcing the importance of motor experience in motor learning.

Our findings related to physical changes corroborate what others have found in terms of changes in endurance, as well as improvements (though not for all participants) in spasticity [24] and bowel function [4]. Our companion paper (Khan et al) describes changes in function and neuroplasticity in more detail. The psychological benefits that others have alluded to anecdotally [4] were discussed at length by our participants. Some of those benefits were related to being able to do everyday things, others talked about the importance of purpose, while many appreciated the social aspect of training. At the interview 2 months after training stopped, in general, participants felt that their mental health was good – though there were two participants in particular who appeared to struggle to maintain a positive outlook after the cessation of training.

Finally, our participants greatly valued their role in knowledge generation. They were happy to give of their time and their body to help learn about and improve a device. Recommendations were provided about the equipment (as conferred in the results) and perceived uses which ranged from walking at home or outside to use in a rehabilitation setting or community recreation centre. The desire to have exoskeletons available for use in a variety of settings, including community centres corroborates findings from a previous study [28] and broadens the number of potential stakeholders for these devices. Despite our participants’ willingness to contribute, manufacturers of exoskeletons as well as rehabilitation professionals must be careful not to exploit the goodwill of persons with disabilities to market devices or engage participants as solely a way to help them walk again [26]. There are a growing number of studies that focus on health and functional benefits [15, 17, 18] and these devices should also be promoted in those more general terms. Nonetheless, it is difficult or impossible to disentangle some of the potential health and functional benefits from the reality that some of these benefits come from the action of standing and walking. These tensions and the risk of reinforcing the ability hierarchy are something that health care providers, researchers, and manufacturers should be aware of.

### Limitations

The sample of persons who participated was largely a group that were unemployed (2 of 11 had jobs), which may have led to the many discussions about having a purpose, a place to go and being able to contribute. Being part of the study involved a huge time commitment and it may be that persons who were employed couldn’t afford the time. Several of our participants had been involved in previous research studies and clearly embraced their role in knowledge acquisition. Because all participants were volunteers we do not know the perspectives of others who were either not eligible or unable to commit to the time required by the study. Nevertheless, the sample was diverse in terms of age, level of injury and time since injury, thereby providing varied perspectives.

## Conclusion

Participants were overwhelmingly positive about the experience of learning to walk with a ReWalk. They saw it as an opportunity to do similar activities as peers, but were also realistic in their ability to use the device on their own. Increasing availability and support for the use of exoskeleton devices in community settings is an interim step to home use as the devices continue to improve. Incorporating the view of the user in the design and refinement of exoskeletons will help ensure that the devices are embraced by future users.

## Data Availability

Audio files and transcripts are not available for review because of the risk of participant identification.

## Abbreviations

SCI: Spinal cord injury
